# Clinical Application of Magnetic Resonance Imaging in the Diagnosis of NAFLD

**DOI:** 10.2174/0115734056383747251124104257

**Published:** 2026-01-13

**Authors:** Jifei Deng, Shizhao Ou, Lijuan Lai, Derek Corica, Lijun Dong, Yujiang Fang, Guojun Song

**Affiliations:** 1 Department of Radiology, Nanhua Hospital Affiliated to Nanhua University, Hengyang, China; 2 Department of Microbiology, Immunology & Pathology, Des Moines University, Des Moines, United States of America; 3 Department of Obstetrics and Gynecology, The First Dongguan Affiliated Hospital, Guangdong Medical University, Dongguan, China

**Keywords:** MRI, Nonalcoholic fatty liver disease, Apparent diffusion coefficient, Liver surface nodules, liver steatosis, liver cell ballooning

## Abstract

**Introduction:**

This review aims to evaluate the accuracy of Magnetic Resonance Imaging (MRI) in diagnosing Non-Alcoholic Fatty Liver Disease (NAFLD) based on the published literature.

**Methods:**

A PubMed search was performed using the keywords NAFLD and MRI, and the literature search deadline was set before April 2025.

**Results:**

A total of 86 studies out of 405 retrieved were included in this study. The results showed that Magnetic Resonance Imaging Proton Density Fat Fraction (MRI-PDFF) was positively correlated with steatosis grading. The proton Density Fat Fraction Magnetic Resonance Spectroscopy (PDFF-MRS) threshold of 5% could be used to diagnose liver steatosis. Apparent Diffusion Coefficient (ADC) of NAFLD patients was significantly lower than that of controls. A 15% increase in Magnetic Resonance Elastography (MRE) was the strongest predictor of progression to advanced fibrosis in NAFLD. The corrected T1 (cT1) cutoff value of 875 ms was used to identify liver fibrosis in NAFLD. The correlation between the Liver Surface Nodules (LSN) score and the stage of fibrosis in NAFLD was very strong. Dynamic enhanced MRI (DCE-MRI) parameters increased with increasing severity of NAFLD and fibrosis.

**Discussion:**

This study evaluated the value of multiple MRI techniques in diagnosing NAFLD, confirming MRI's high accuracy and reliability as a non-invasive tool for quantifying NAFLD. However, future technical specification harmonization is needed to enhance comparability of results and validate generalizability through multicenter studies.

**Conclusion:**

MRI is a highly reliable and accurate method for diagnosing NAFLD.

## 1 INTRODUCTION

Non-Alcoholic Fatty Liver Disease (NAFLD) is a chronic liver disease caused by factors other than alcohol and other clearlydefinedliverinjuries.The full histologic spectrum of NAFLD includes liver steatosis, lobular inflammation, liver cell ballooning, and fibrosis. NAFLD can progress to Non-Alcoholic Steatohepatitis (NASH) and eventually develop varying degrees of liver cirrhosis. NAFLD varies significantly among different regions and populations. In terms of geographical distribution, the prevalence is highest in North America and the Middle East. Saudi Arabia and the United States are particularly prominent in this regard. Among Asian regions, China has the highest prevalence of NAFLD. The prevalence of NAFLD has increased globally over the past few decades, and it is currently estimated that approximately 25% of adults have NAFLD [1]. NAFLD is associated with other metabolic syndrome disorders such as type II diabetes, dyslipidemia, and cardiovascular disease. Early detection and treatment can help slow or potentially reverse the progression of NAFLD [2].

According to the NAFLD standardized histological scoring system, liver steatosis is classified into 4 grades, ranging from 0 to 3 [3]. Histologic analysis accurately assesses steatosis in NAFLD, but liver biopsies are invasive, few patients are willing to undergo repeat biopsies to monitor disease progression and assess response to therapy, and liver biopsies may have risks of causing serious complications and sampling errors [4]. Patients with NAFLD may show elevated levels of indicators such as triglycerides, total cholesterol, Alanine Aminotransferase (ALT), Aspartate Aminotransferase (AST), and Gamma-Glutamyl Transpeptidase (GGT). However, these indicators lack specificity, and abnormalities may also occur in other liver diseases. In recent years, NAFLD evaluation has also been performed using a variety of imaging modalities, including Ultrasound (US), Computed Tomography (CT), and multimodal MRI sequences. US is a rapid and noninvasive imaging modality for assessing liver stiffness and steatosis. However, the diagnostic accuracy of US in detecting NAFLD may be influenced by various factors, such as age, ascites, Body Mass Index (BMI), visceral fat, and rib gap, resulting in less accuracy [5]. CT may detect liver fat, but it is not recommended due to its low sensitivity and radiation exposure. Compared with ultrasound and CT, MRI can provide a more precise and quantitative assessment of the liver fat content. The non-invasive nature of this technology makes it more attractive in clinical applications, especially for patients who require long-term monitoring. During treatment, MRI can provide a detailed, accurate evaluation of changes in the liver. Its multi-parametric imaging function enables quantitative analysis of liver fat content over time, allowing clinicians to directly observe the reduction in fat deposition as the treatment progresses. This is much more reliable than a subjective, less accurate ultrasound. In addition, MRI offers high soft-tissue resolution and can detect improvements in relevant liver tissue changes, such as the alleviation of inflammation or the reversal of early-stage liver fibrosis, thereby providing a comprehensive perspective on treatment response. Unlike CT, MRI does not generate ionizing radiation, so patients can undergo multiple scans without incurring cumulative risks associated with radiation exposure, enabling continuous and safe monitoring of treatment effects throughout the entire treatment management process for NAFLD.

MRI is a noninvasive method to evaluate NAFLD [6]. However, its accuracy in the diagnosis of NAFLD has not been well documented. By reviewing the published data, this study is designed to assess the accuracy of MRI techniques in diagnosing NAFLD, using liver histology as the gold standard.

## 2 MATERIALS AND METHODS

Studies on the diagnostic accuracy of magnetic resonance imaging for NAFLD steatosis were retrieved, and the data from all included studies were analyzed. Study selection is reported in Fig. (**1**).

A PubMed search was performed using the keywords NAFLD and MRI, and the literature search deadline was set before April 2025. The search results were first screened based on the titles and abstracts of the manuscripts, and the full texts were analyzed in this study. This review focuses not only on studies of the diagnostic accuracy of MRI techniques for the diagnosis of steatosis in NAFLD using liver histology as a reference standard, but also collects studies of MRI for the assessment of the progressive state of NAFLD, including NASH and fibrosis.

Several types of studies were excluded from our review, including studies on the diagnosis of NAFLD that used MRI as a gold standard to assess the accuracy of other imaging methods, and studies for which the full text was not available.

This study evaluated the studies based on the classification of magnetic resonance imaging techniques. Then, we collected information on the sensitivity, specificity, diagnostic accuracy, Area Under the Subject Operating Characteristic Curve (AUROC), 95% Confidence Intervals (95% CI), cutoff value, Positive Predictive Value (PPV), and Negative Predictive Value (NPV) of MRI for the diagnosis of NAFLD. Finally, all the collected data were summarized and analyzed (Table **1**).

## 3 RESULTS

### 3.1 Magnetic Resonance Imaging Proton Density Fat Fraction (MRI-PDFF) Positively Correlates with Liver Steatosis Grading

Fat is stored as triglycerides in the liver, so steatosis is best measured by the number of molecules of triglycerides [7]. MRI-PDFF is defined as the fraction of mobile protons in triglycerides relative to those in water. It has been proposed as a standardized MRI biomarker of steatosis [8]. It is worth noting that liver histology assesses the number of fat cells in the liver, whereas MRI-PDFF estimates the overall percentage of fat molecules in the liver as a fraction of MRI-visible protons. Since cellular steatosis is usually no more than 50%, the MRI-PDFF percentage is almost always less than half of the value obtained by histology [9]. For MRI-PDFF measurements, several MRI techniques are available, such as iterative decomposition of water and fat with echo asymmetry and least-squares estimation (IDEAL-IQ), volume-interpolated breath-hold examination Dixon (VIBE Dixon), multi-echo two-point Dixon Quant (mDixon Quant), and other techniques [8].

MRI-PDFF is strongly associated with liver steatosis and can be used to differentiate among varying degrees of steatosis [10-13]. MRI-PDFF thresholds for histologic steatosis grading in NAFLD are 6.4%-8.9% for grade 1, 16.3%-17.4% for grade 2, and 22.1%-25.0% for grade 3, respectively [14, 15]. Tang *et al*. also demonstrated that MRI-PDFF was positively correlated with steatosis grading (r = 0.69; 95% CI: 0.50,0.81; *P* < 0.001) and that MRI-PDFF was highly specific for steatosis grading and was not affected by other histologic or clinical parameters, including age, gender, BMI, lobular inflammation, liver cell ballooning, fibrosis and magnetic field strength [16, 17]. Classification of steatosis into grades 0 and 1 according to the 6.4% MRI-PDFF cutoff reported above resulted in a sensitivity of 68%, a specificity of 98%, and an AUROC of 0.82. The cutoffs for grades 2 and 3 were 16.3% and 21.7%, respectively, and the AUROCs were 0.95 and 0.96, with sensitivities of 83% and 84%, and a specificity of 90%. MRI-PDFF correlated with histologic steatosis grading and accurately classified steatosis grading longitudinally [9, 18]. A 5.15% decrease in MRI-PDFF identified a decreased grade of steatosis with 90% specificity and 58% sensitivity, whereas a 5.6% increase in MRI-PDFF identified an elevated grade of steatosis with 90% specificity and 57% sensitivity [18]. In another study examining the degree of MRI-PDFF reduction relative to histologic response, a 29% relative reduction in MRI-PDFF was associated with lower steatosis grade in NASH [19].

In a study exploring the relationship between liver steatosis and fibrosis progression, Ajmera *et al*. [20] showed that increased liver fat content as measured by MRI-PDFF was associated with fibrosis progression. Tamaki *et al*. [21] further demonstrated that a ≥30% reduction in MRI-PDFF can be estimated to represent an improvement in fibrosis to ≥ stage 1. In addition, MRI-PDFF combined with clinical data was highly accurate in differentiating NASH from fibrosis (AUROC: 0.93; 95% CI: 0.88-0.97), with a cutoff value of 0.242 corresponding to a sensitivity of 75.0%, a specificity of 90%, a PPV of 50.0%, and an NPV of 96.5%, whereas a cutoff value of 0.165 corresponded to a sensitivity of 90% and specificity of 72.2%, PPV of 29.4% and NPV of 98.1% [22]. Thus, MRI-PDFF can be used as a dynamic, more sensitive imaging biomarker than liver biopsy [23].

### 3.2 Magnetic Resonance Spectroscopy (MRS) can Quantify Liver Steatosis

MRS is an MRI imaging technique that uses chemical shifts to perform MRI spectral scans to analyze the structure and properties of substances. Hydrogen Proton Magnetic Resonance Spectroscopy (^1^H-MRS) quantifies liver metabo-lites, especially liver fat deposition. ^1^H-MRS is obtained based on short echo time (TE) and long TE. Short TE ^1^H-MRS provides insight into fat accumulation in NAFLD. Unlike short TE ^1^H-MRS, long TE ^1^H-MRS is better able to distinguish longer T2 relaxation metabolites such as alanine and lactate [24]. To further explore the diagnostic value of long TE ^1^H-MRS for NAFLD, Kim *et al*. [25] demonstrated that the application of long TE ^1^H-MRS is potentially valuable for the quantitative diagnosis of NASH and simple fat accumulation, and that alanine, as well as lactate, are positively correlated with the degree of steatosis.

It has been shown that the Proton Density Fat Fraction Magnetic Resonance Spectroscopy (PDFF-MRS) is the most accurate non-invasive imaging technique for liver fat quantification [26]. PDFF-MRS is defined as the ratio of area under the methylene (1.3ppm) peak to area under the methylene (1.3ppm) peak + area under the water peaks. PDFF-MRS correlated strongly with MRI-PDFF (r = 0.983-0.99, *P* < 0.001), and, as with MRI-PDFF, PDFF-MRS also showed a strong correlation with steatosis grading [27-30]. Eddowes *et al*. [26] showed that the median PDFF-MRS for NAFLD grade 1, 2, and 3 steatosis was 0.3%, 11.3%, 23.7%, and 31.5% (*P* < 0.001), respectively. The findings of Di Martino *et al*. [31] suggested that a PDFF-MRS cutoff value of 5% can be used to diagnose liver steatosis in adolescents and adults; however, when MRI-PDFF is used to quantify liver steatosis, a cutoff value of 3.5% is more appropriate. Furthermore, in their experience, MRS showed excellent correlation with liver biopsy results.

In addition, phosphorus magnetic resonance spectroscopy (^31^P-MRS) allows in vivo assessment of liver cell metabolism and shows potential for biochemical analysis in liver disease. The ratio between ^31^P-MRS-derived Nucleotide Triphosphate and Triphosphate (αNTP/TP) reflects cellular energy depletion and concentrations of specific metabolites and can be used to diagnose the severity of NAFLD. The results show that the AUROC of αNTP/TP for diagnosing NAFLD was 0.71. The sensitivity of the diagnosis of NASH was 91% when the cutoff value of αNTP/TP was 16.36%, and the specificity was 91% when the cutoff value was 10.57%.^31^P-MRS shows significant changes in different NAFLD states and has high diagnostic accuracy for NASH [32].

### 3.3 Diffusion-weighted Imaging (DWI) has Some Value in the Diagnosis of NAFLD

DWI can be used to qualitatively and quantitatively analyze the random Brownian motion of water molecules and helps to detect changes in liver tissue structure [33]. DWI may replace invasive diagnostic procedures in preclinical studies, clinical trials, and routine exams, given the growing need to develop non-invasive imaging biomarkers [34].

Over the past decade, DWI has shown potential benefits in diagnosing liver disease, and it has been proposed that fatty deposits within and between liver cells reduce interstitial space and decrease molecular diffusion. Makhija *et al*. [35] showed that although DWI was not accurate in grading liver steatosis in patients with NAFLD, a significant increase in liver fat deposition lowered the Apparent Diffusion Coefficient (ADC) values, which were 1.30 ± 0.21 x 10^-3^ mm^2^/s for mild fatty livers (with a fat fraction of 5-10%), and 1.09 ± 0.21 x 10^-3^ mm^2^/s for severe fatty livers (with a fat fraction of 20-35%). This is similar to the study of Poyraz *et al*. [36], where ADC values were measured by taking b = 0 mm^2^/s and 1000 mm^2^ /s. The mean ADC in the NAFLD group was 1.20 ± 0.22 × 10^-3^ mm^2^/s, significantly lower than that in the control group of 1.32 ± 0.23 × 10^-3^ mm^2^/s (*p* =0.02), and there was a negative correlation between the ADC values and steatosis grading (r = -0.39, *p* < 0.0001). Manning *et al*. [37] also showed that ADC decreased with increasing grades of steatosis (*p* < 0.05). In a study applying DWI to assess liver fibrosis in patients with NAFLD, the results showed a significant correlation between fibrosis staging and ADC when taking a b-value of 0 ~ 1000 s/mm^2^ (r = -0.57, *P* = 0.01). For patients who took a b value of 0-1000 s/mm^2^ to diagnose stage ≥ 3 fibrosis, the AUROC was 0.93 and the optimal ADC cutoff was 1.16 × 10^-3^ mm^2^ /s [38].

### 3.4 Magnetic Resonance Elastography (MRE) is an Accurate Method for Diagnosing Liver Fibrosis in NAFLD

MRE is the use of an externally driven system to create mechanical waves that propagate through the body and generate quantitative images that show the shear modulus of the tissue. MRE is a valid and accurate noninvasive biomarker for diagnosing NAFLD [39], which is analyzed using shear waves induced in the liver by low-frequency abdominal vibrations [7]. During MRE acquisition, vibration is applied continuously, typically in the range of 20 Hz to 500 Hz [40]. MRE is highly accurate in detecting and grading liver fibrosis [29-41]. Studies show that MRE is both highly accurate for detecting liver fibrosis with low inter-observer variability and is not affected by patient demographics [42].

A growing number of studies have shown that MRE is an accurate method for diagnosing and staging liver fibrosis in NAFLD [43]. According to the Mayo Clinic panel's recommendations, the following are the reference thresholds for liver fibrosis: normal < 2.5 kPa, normal or inflammatory 2.5 to 2.9 kPa, stage 1 to 2 fibrosis 2.9 to 3.5 kPa, stage 2 to 3 fibrosis 3.5 to 4 kPa, stage 3 to 4 fibrosis 4 to 5 kPa, and stage 4 fibrosis/cirrhosis >5 kPa [44]. The AUROC for MRE to differentiate stage 0-2 fibrosis from stage 3-4 fibrosis was 0.924 (*P* < 0.0001), The threshold >3.63 kPa had a sensitivity of 0.86 (95% CI: 0.65-0.97), a specificity of 0.91 (95% CI: 0.83-0.96), a positive predictive value of 0.68 (95% CI: 0.48-0.84), and a negative predictive value of 0.97 (95% CI: 0.91-0.99) [45]. Another study demonstrated the high accuracy of MRE, with sensitivities and specificities for diagnosing ≥ 1, ≥ 2, ≥ 3, and = 4 stages of fibrosis of 0.87 (95% CI: 0.84-0.89) and 0.92 (95% CI: 0.87-0.96), 0.87 (95% CI: 0.84-0.90), and 0.92 (95% CI: 0.89- 0.95), 0.88 (95% CI: 0.85-0.91) and 0.91 (95% CI: 0.88-0.93), 0.91 (95% CI: 0.87-0.94), and 0.92 (95% CI: 0.89-0.94), and AUROCs for MRE diagnosis of stage >1, >2, >3, and 4 fibrosis were 0.86-0.9502, 0.87-0.9663, 0.90-0.9644, and 0.91-0.9768, and the optimal MRE cutoff values were 2.61 kPa, 2.97 kPa, 3.62 kPa, and 4.69 kPa, respectively [46-48]. A longitudinal study by Ajmera *et al*. [49] showed that a 15% increase in MRE was the strongest predictor of progression to stage 4 fibrosis (95% CI: 1.35-17.84; *P* = 0.0159). Reduced Liver Stiffness (LSM) measured by MRE was also predictive of improved fibrosis stage, with an AUROC of 0.62 (95% CI: 0.46-0.78), and the optimal threshold was a relative reduction of ≥ 0%, at which MRE had a sensitivity of 67%, specificity of 64%, positive predictive value of 48%, and negative predictive value of 79% [7]. LSM has been shown to outperform other non-invasive biomarkers and tests for fibrosis staging [50]. In addition, the sensitivity and specificity of MRE, when combined with laboratory findings, for the diagnosis of NAFLD are further improved [39].

### 3.5 Corrected T1 (cT1) can Assess NAFLD after Removing the Effects of Iron

cT1 is the magnetic resonance imaging parameter obtained by removing the effect of iron from the T1 sequence. cT1 is a new quantitative MRI indicator for assessing liver inflammation and fibrosis that has been shown to diagnose NAFLD [51]. However, the accumulation of excess iron in the liver may be a confusing factor. The presence of iron in the liver increases the magnetization rate and shortens the T2* relaxation time of the tissue due to the increase in the local magnetic field, so that T2* is negatively correlated with the iron content of the liver [52-54]. To correct for the effects of iron, the T1 and T2* data can be combined by a proprietary algorithm to obtain cT1 [55].

Normal T1 values are 550-620 ms for 1.5 T MRI and 700-850 ms for 3 T MRI [56]. In a recent study, a reference range of cT1 values in healthy populations was determined to be 573-852 ms, with a median cT1 value of 666 ms (95% CI 600-763 ms) [51]. cT1 has been used as an endpoint in several clinical studies to evaluate different stages of liver disease and to monitor response to therapy [57]. The AUROC (95% CI) for cT1 in differentiating patients with simple steatosis from those with NASH was 0.69 (0.50-0.88) [26]. Dennis *et al*. [58] showed that cT1 was positively correlated with inflammation and fibrosis. Some investigators have suggested an optimal cT1 cutoff value of 875 ms to differentiate patients with NASH or fibrosis, with a sensitivity and specificity of 97% and 50%, respectively. The findings of Dennis *et al*. [58] also showed a positive correlation between cT1 and all histopathological features of NASH. This demonstrates that increased liver steatosis is associated with more severe liver cell injury [59].

### 3.6 Liver Surface Nodules (LSN) have a Strong Correlation with Liver Fibrosis Staging in Patients with NAFLD

LSN is a new method for quantitative measurement of liver surface irregularities. Computerized post-processing techniques define nodules as the degree of deviation of the liver surface from the fitted polynomial line, allowing objective quantification of progressive liver remodeling, scarring, and regenerative nodules [60]. Liver remodeling in NAFLD is usually micronodular, and morphological changes may be subtle on conventional cross-sectional imaging. MRI-based nodularity scoring of the liver surface is a promising, noninvasive, and objective tool for rapidly and accurately detecting different stages of fibrosis in patients with NAFLD.

It has been shown that the difference in LSN scores between different fibrosis stages in NAFLD was statistically significant (*p* < 0.001), and the correlation between LSN scores and fibrosis staging was also strong (ρ = 0.71; *p* < 0.001). The AUROC for detecting stage 3 and 4 fibrosis was 0.80 (95% CI: 0.66-0.95) and 0.86 (95% CI: 0.75-0.97), with thresholds of 2.23 and 2.44, respectively. The sensitivity of this method for detecting stage 4 fibrosis was 81%, and the specificity was 88% [61]. A study by Dioguardi *et al*. [2] also showed that MRI-LSN has a good diagnostic value for NAFLD stage 4 fibrosis. The AUROC for diagnosing stage 4 fibrosis with MRI-LSN ≥ 2.50 was 0.838 (95% CI: 0.767-0.894, sensitivity 67.3%, specificity 88.9%, positive predictive value 77.8%, negative predictive value 82.5%).

### 3.7 Dynamic Enhanced MRI (DCE-MRI) is an Excellent Diagnostic Indicator for Fibrosis in Patients with NAFLD

DCE-MRI performs magnetic resonance scanning simultaneously with rapid contrast injection to obtain dynamic information on the distribution of contrast in the capillary network and the interstitial space, thereby reflecting changes in microcirculation, perfusion, and capillary permeability of the lesion. DCE-MRI provides several semiquantitative parameters for measuring liver perfusion, including the intravascular-to-extravascular interstitial transport coefficient (Ktrans), extravascular cell volume fraction (Ve), and plasma volume fraction (Vp). Ktrans describes the transcellular transport of low-molecular-weight contrast agents, which is determined by three main factors: blood perfusion, transvascular transport, and diffusion within the interstitium. According to the principles of quantitative DCE-MRI, the small-molecule, non-specific GdDTPA-BMA is more suitable for the calculation of pharmacokinetic parameters [62]. Ktrans is an excellent diagnostic index for distinguishing normal liver from NAFLD fibrosis [63].

DCE-MRI significantly correlates with NAFLD and helps identify NASH [64]. The full histologic spectrum of NAFLD includes steatosis, lobular inflammation, liver cell ballooning, and fibrosis. One possible reason for the increase in Ktrans is the infiltration of inflammatory cells into the liver lobules. In addition, elevated liver sinusoidal pressure from various causes can accelerate the transfer of blood from the liver sinusoids to the Extracellular Extravascular Space (EES) [65]. Wu *et al* [62] demonstrate that Ktrans increases with increasing NAFLD severity and fibrosis. Ktrans distinguishes between the control and NASH groups with an AUROC of 0.968 and between the normal and overall fibrosis groups with an AUROC of 0.898. Thus, DCE-MRI may have a role in assessing NAFLD severity.

## 4 DISCUSSION

This study provides strong evidence that MRI is a highly accurate, noninvasive method for diagnosing NAFLD. Our study is consistent with previous studies showing that MRI-PDFF, MRS, and DWI can quantify liver steatosis, and that MRE, cT1, LSN, and DCE-MRI are excellent diagnostic indicators for fibrosis in patients with NAFLD. Our study is unique in that it focuses primarily on evaluating the diagnostic value of MRI for NAFLD and assesses a broader range of MRI techniques. Furthermore, our study not only evaluates steatosis in patients with NAFLD but also assesses their progressive state of NASH and fibrosis.

In fact, most studies are consistent with ours, showing that MRI-PDFF is currently the most accurate and reproducible method for non-invasive quantification of liver fat [42, 66-71]. It has even been suggested that longitudinal changes in MRI-PDFF are more sensitive than biopsy for detecting fatty changes in the liver [72-74]. However, Wildman-Tobriner *et al*. [75] showed that MRI-PDFF was not ideal for identifying patients with stage 3 and 4 fibrosis. Therefore, liver biopsy analysis remains necessary. Similar to this review, Makhija *et al*. showed that MRS could accurately quantify and monitor liver fat content [12]. However, it has also been shown that ^31^P MRS does not detect significant fibrosis, perhaps because it is not suitable for distinguishing the degree of liver fibrosis. In addition, MRS has some limitations: long acquisition time, technical demands, limited spatial coverage, inability to assess the full extent of liver steatosis, and a tendency to lead to sampling errors. This demonstrates that at the current moment, MRS is not a viable substitute for liver biopsy. However, the above-mentioned limitations of MRS can be addressed in the future by optimizing the scanning sequence and enhancing the professional skills of the scanning operators. Most studies show that DWI shows some value in the diagnosis of NAFLD. However, other studies show that DWI does not help differentiate steatosis from liver fibrosis, which may be due to the low b-values in these studies [76, 77]. Another reason could be that ADC in NAFLD patients is affected by iron deposition [78]. Some researchers suggest that 3 T DWI with a b value of 1,000 s/mm^2^ has a relative advantage for assessing liver fibrosis in patients with NAFLD [38].

A growing body of research suggests that MRE can more accurately and quantitatively assess NASH and liver fibrosis [39, 43, 79]. However, Chen *et al*. [80] showed that there was no significant difference in LSM across steatosis grades within fibrosis stages. In a study by Goyal *et al*. [81], it was even concluded that MRE did not change significantly in NASH patients after 12 weeks of standard treatment. Additionally, MRE mechanical properties did not significantly correlate with steatosis, liver cell edema, or NASH [82]. Goyal *et al*. suggested that the negative results could be due to the MRE parameters not being measured multiple times in their study, resulting in low accuracy. The susceptibility of liver biopsy itself to sampling error may be one of the reasons for negative results. Our study shows the high sensitivity of cT1 for the diagnosis of NAFLD. However, cT1 is sensitive to steatosis, ballooning, inflammation, and fibrosis, and therefore cannot be used as a pure biomarker of steatosis [83]. Studies indicate that although cT1 does not accurately diagnose steatosis, cT1 can only be used to differentiate between patients with NAFLD and patients with NASH. In the study by Eddowes *et al*. [26], cT1 did not correlate significantly with liver inflammation and stiffness and could not be used for liver fibrosis staging. Whereas the combination of cT1, glutamate dehydrogenase (AST), and fasting blood glucose levels can identify NASH with fibrosis, identifying the ideal combination of biomarkers will be the focus of further studies [29]. Non-enhanced T1-weighted MRI-derived LSN can be used as a quantitative imaging biomarker of liver injury. However, Besa *et al*. [84] suggested that MRE may outperform LSN in quantifying liver fibrosis. However, LSN measurements can be performed quickly and retrospectively on MR images from a single sequence in less than a minute, without the need for specific acquisitions, thereby greatly increasing the utility of LSN [85]. The conclusions of the current studies on DCE-MRI for the diagnosis of NAFLD are generally similar to those of the review, but concerns about the safety of the contrast agent make DCE-MRI less suitable as a routine diagnostic method.

Although liver biopsy is necessary, diagnosing NAFLD with multimodal magnetic resonance imaging can significantly reduce costs compared to liver biopsy alone. Some researchers [86] have pointed out that using only cT1 with a threshold of 875 milliseconds for the examination of all patients, rather than performing biopsies on all of them, is estimated to save 150,218 pounds for every 1,000 patients. Applying liver stiffness measurement (with a threshold of 7.0 kilopascals) can reduce costs by 307.92 pounds compared to liver biopsy. Moreover, combining multiple MRI techniques is likely to result in cost savings compared to biopsies.

Compared with the systematic review by Garg *et al*. (2025) in Cureus [86], although this study on NAFLD diagnosis overlaps with this article in terms of theme, there are significant differences in research design, methodology, and clinical focus. This study evaluated the diagnostic efficacy of various non-invasive biomarkers (including serum markers, imaging examinations, and composite scoring systems) from 2010 to 2024. It was found that technologies such as MRI-PDFF and MRE have excellent diagnostic accuracy (AUC 0.81-0.90), but it was also noted that some biomarkers, such as NIS4, have insufficient specificity (only 63%). In contrast, this study focuses on the diagnostic value of MRI technology and conducts a detailed analysis of the performance characteristics of seven MRI technologies, including MRI-PDFF, MRE, MRS, cT1, DCE-MRI, LSN, and DWI, and provides specific diagnostic thresholds (such as the critical values for PDFF classification (6.4%-25.0%)). The two studies also differ in their clinical application recommendations: Garg's study emphasizes the public health value of non-invasive tools for reducing liver biopsies and recommends MRI-PDFF and MRE for fat quantification and fibrosis staging, whereas this study focuses more on radiology practice and proposes actionable standards for multi-parametric MRI technology. Overall, in clinical practice, the advantages of the two studies can be combined: refer to serum markers or ultrasound examinations during the initial screening and adopt a multi-parametric MRI protocol for accurate staging, thereby achieving accurate diagnosis and hierarchical management of NAFLD.

It is necessary to point out that our study has some limitations: many studies applied multiple magnetic resonance imaging methods for the diagnosis of NAFLD, which were not evaluated together in this article; instead, the different imaging techniques were described separately, undermining the integrity of the original article. The diagnostic capabilities of ultrasound and MRI were compared in several other studies, but the comparative data were not presented because they were beyond the scope of this review, which reduces the evidence that MRI is the most accurate technique for diagnosing NAFLD.

## CONCLUSION

In conclusion, although MRI is not yet a substitute for pathological study. It remains a highly accurate, noninvasive technique for diagnosing nafld. Such a study may help demonstrate the usefulness of mri as a powerful diagnostic tool for patients with NAFLD. Future research will primarily explore the clinical utility of integrated diagnostic models incorporating serum iron and mri in the accurate stratification of NAFLD.

## AUTHORS’ CONTRIBUTIONS

The authors confirm contribution to the paper as follows: G.S.,Y.F.,L.D.: Study conception and design; J.D.: Data collection, S.O.: Data Analysis or Interpretation; L.L.: Methodology; D.C.: Investigation; J.D.: Draft manuscript. All authors reviewed the results and approved the final version of the manuscript.

## Figures and Tables

**Fig. (1):**
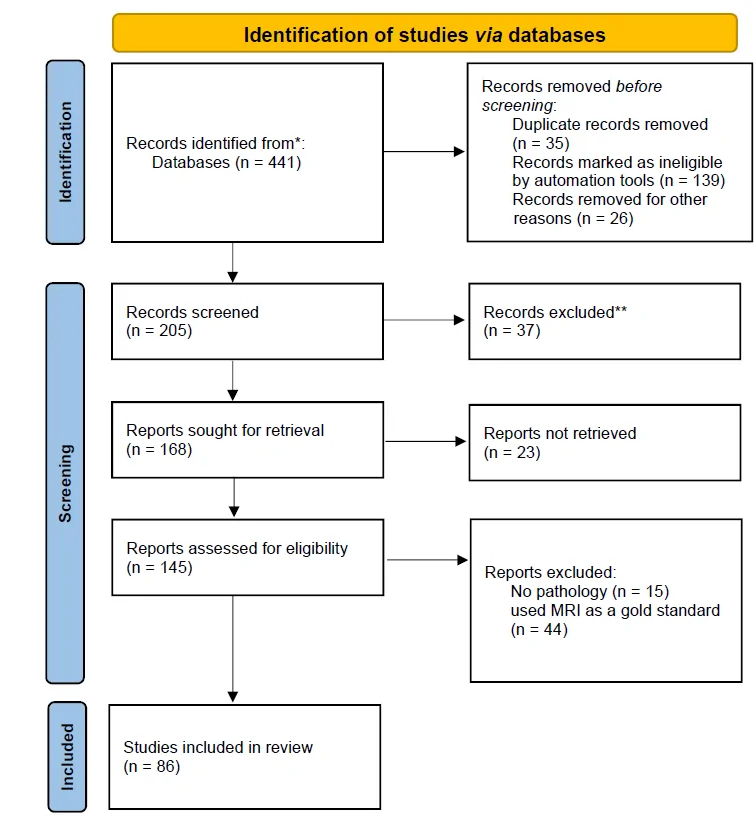
Study selection.

**Table 1:** Studies using one or more MRI imaging techniques as diagnostic of NAFLD.

**Study**	**Research Design and Population**	**technology**	**Research Index**	**Research result**
[2]	Retrospective 142 patients	1.5-T LSN	LSN	LSN≥2.50 AUC=0.838(95%CI 0.767-0.8) specificity =88.9%,PPV=77.8%,NPV=82.5%
[6]	Prospective 47 patients	3T MRS,MRE T1 Mapping	FF,LS, T1	FF,LS,T1 AUC = 0.870,0.951 , 0.664
[7]	Prospective 72with NASH	MRE MRI	PDFF LSM	MRE AUROC=0.62 (95% CI 0.46-0.78),sensitivity= 67%, specificity= 64%,PPV =48%,NPV =79%.MRI-PDFF AUROC= 0.70 (95% CI 0.57-0.83),sensitivity= 89%,specificity =47%,PPV 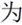 =39%,NPV=92%
[9]	Prospective 169 children	1.5T 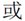 3T MRI	PDFF	grade 1 AUROC= 0.87(95% [CI]:0.80, 0.94),grade 2-3 AUROC =0.79(0.70, 0.87).
[11]	Prospective 160 patients	1.5T MRE	Liver volume	Liver volume(Odds ratio = 1.001;95% CI,1.000–1.002;p = 0.007)
[12]	Prospective 41 patients	1.5 T MRS	FSF PDFF	FSF(19.30 ± 9.09% - 11.18 ± 7.61%;P < 0.05), MRS-PDFF(18.79 ±8.53% -10.64 ± 6.66%)
[13]	Retrospective 63 children	3.0 T MRI	HFF	HFF related to Each steatosis grade=10.4%,23.7% and 31.1%,P < 0.001
[18]	Multicenter, randomized, double-blind 113 patients	1.5Tor3T MRI	PDFF	grade 0-1 vs grade 2-3, AUROC=0.95 (95% CI, 0.91-0.98),grade 0-2 vs grade 3,AUROC=0.96 (95% CI, 0.93-0.99)
[19]	Secondary longitudinal analysis 50 patients	MRI	PDFF	MRI-PDFF decline of 29% related to Histological response of NASH
[20]	Prospective 95 patients	3T MRI	PDFF	The proportion of patients with fibrosis progression in the high liver fat group (MRI-PDFF ≥ 15.7%) was higher (38.1%) than that in the low liver fat group (11.8%) (P= 0.067)
[21]	Prospective 100 patients	3T MRI	PDFF	PDFF Dominance ratio=6.46(1.1-37.0, p = 0.04)
[22]	Retrospective 347 patients	3T MRE MRI	MAST mark	MAST mark AUC= 0.93;95% (0.88-0.97)
[23]	Prospective 50 patients	3T MRI	PDFF,TLV	A 10% reduction in MRI-PDFF predicted a 257 mL reduction in TLV
[26]	Prospective 56 patients;50 with NAFLD	3.0T MRI, MRS T1,T2*	PDFF cT1◦	cT1 AUROC (95% CI) =0.69(0.50-0.88);PDFF-Dixon,PDFF-MRS AUROC= (95% CI) =1.00(1.00-1.00)
[27]	Retrospective 47 patients	3T MRI,MRS	PDFF	≥steatosis grade 2,≥steatosis grade 3 MRI-PDFF AUROC =0.846 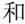 0.855.≥steatosis grade 2,≥steatosis grade 3 MRS AUROC =0.860,0.878.
[28]	Retrospective 19 patients	1.5 T MRI MRS	PDFF	MRI-PDFF closely correlated with histological steatosis (r = 0.743, P < 0.001), MRS-PDFF closely correlated with histological steatosis (r = 0.712, P < 0.00
[29]	Prospective 37with NAFLD	3.0 T MRE	LSN	AUC= 0.79
[30]	Prospective 13 patients	1.5T MRI MRS	MRI-PDFF; MRS-PDFF	MRI-PDFF excellent correlation with MRS-PDFF (r=0.984;CI: 0.978 ~ 0.989),PDFF Strong correlation with histology (r=0.850;CI: 0.791-0.894)
[37]	Prospective 64 children	3T DWI	ADC,D,F	ADC:1.3 (0.94 –1.8) x 10−3 mm^2^ /s; D: 0.82 (0.56 – 1.0) x 10−3 mm^2^ /s; F: 17 (6.0 – 28) %
[38]	18 patients	3 T DWI	ADC	AUC= 0.93, cut-off value= 1.16 ×10-3 mm^2^ /s
[39]	Prospective 127 patients	3 T MRE	LSM	MRE accuracy =0.841;95% CI 0.772–0.910)
[43]	90 patients	1.5 T MRE	LSC_N10,GUCI	F2-4:MRE,LSC_N10,GUCI AUROC=0.78,0.69,0.78,F3-4:AUROC =0.93,0.81,0.82
[48]	Cross-sectional study,104 patients	3T MRI MRE	MRI-PDFF LSN	MRE:fibrosis 1 -4 AUROC =0.82(95% CI,0.74-0.91),0.89(95% CI,0.83-0.96),0.87(95% CI,0.78-0.96), 0.87(95% CI,0.71-1.00);MRI-PDFF: Grade 1-3 steatosis,AUROC = 0.99(95% CI,0.98-1.00),0.90(95% CI,0.82–0.97), 0.92(95% CI,0.84–0.99)
[52]	Prospective 51 patients	3 T T2*	Iron content	sensitivity =76%,specificity =75%,ROC=0.78
[53]	Prospective ,Retrospective 370 patients	3TMRI IDEALIQ	R2* (1/T2*)	R2* AUROC=0.774,cut-off value = 62.5 s ^-1^ .R2* Mapping AUROC =0.799,cut off= 58.5 s ^-1^
[58]	Retrospective 264 patients	3T T1 MRS	cT1和PDFF	cT1 positively correlated with histological steatosis (r = 0.54, P < 0.001), PDFF positively correlated with histological steatosis (r = 0.68, P < 0.001)
[61]	Retrospective 58 patients	1.5 t MRE	LSN	AUROC= 0.86 (95% CI 0.75-0.97), cut-off value =2.44. sensitivity =81%,specificity =88%
[66]	Prospective 152 patients	1.0T MRI	PDFF	steatosis >5%,steatosis S>33%,steatosis >66%,NASH, MRI-PDFF AUROC =0.97,0.95,0.92,0.84
[67]	Case-control study 25 children with NAFLD	1.5 t MRI	HFF	HFF 2% ~ 44%[ average19.0% (95% CI, 15.1 ~ 27.4)], HFF≥5% sensitivity =95.8%
[68]	Retrospective 163 patients	3.0T MRI	PDFF	AUROC (0.81-1.00)
[69]	Cross-sectional study 133 patients	3-T MRI MRE	MRI-PDFF	MRI-PDFF positively correlated with histological steatosis (r=0.978, P<0.001)
[70]	142 patients	3.0T MRE MRI	PDFF LSN	MRE AUROC=0.91(95% ci, 0.86-0.96) (p =.001), PDFF AUROC=0.90 (95% CI, 0.82-0.97) (P< 0.001)
[71]	Retrospective 581 patients	1.5T , 3T MRI	PDFF	Grade 1-3 steatosis AUROC = 1.00 (95% CI, 0.99–1.0),0.77 (95% CI, 0.73–0.^82^], 0.81 (95% CI, 0.76–0.87)
[80]	Retrospective 256 patients	1.5T(MRI)(MRE)	PDFF,LSM	PDFF accuracy> 0.82. There were no significant differences in LSM between all steatosis levels
[81]	Prospective 15 teenagers	3T MRI MRE	PDFF ,LSN	PDFF ,LSN remained stable for 12 weeks
[82]	Retrospective 88 patients	3T MRE	|G*|,G”	|G*|: G″, 2D MRE =2.58±0.61:2.27±0.29 kPa, P = 0.04,3D MRE= 0.42±0.12:0.36±0.11 kPa, P = 0.04

**Abbreviations:** Liver fat fraction (HFF); enhanced hepatic fibrosis (ELF) ;Hepatic surface nodular (LSN); diffusability (D) ;Perfusion fraction (F); Liver fat signal fraction (FSF) ;Average shear stiffness (|G*|); loss modulus (G “) ;Liver hardness (LS); Fat fraction (FF); total liver volume (TLV);Normal liver-spleen ratio (LSC_N10) ;Serum Fibrosis Algorithm (GUCI).

## LIST OF ABBREVIATIONS

NAFLD: Non-Alcoholic Fatty Liver Disease
NASH: Non-Alcoholic Steatohepatitis
MRI: Magnetic Resonance Imaging
MRI-PDFF: Magnetic Resonance Imaging Proton Density Fat Fraction
MRS: Magnetic Resonance Spectroscopy
PDFF-MRS: Proton Density Fat Fraction Magnetic Resonance Spectroscopy
DWI: Diffusion-Weighted Imaging
ADC: Apparent Diffusion Coefficient
MRE: Magnetic Resonance Elastography
cT1: Corrected T1
LSN: Liver Surface Nodularity
DCE-MRI: Dynamic Contrast-Enhanced Magnetic Resonance Imaging
AUROC: Area Under the Receiver Operating Characteristic Curve
CI: Confidence Interval
PPV: Positive Predictive Value
NPV: Negative Predictive Value
IDEAL-IQ: Iterative Decomposition of Water and Fat with Echo Asymmetry and Least Squares Estimation Quantification Sequence
VIBE Dixon: Volume Interpolated Breath-hold Examination Dixon
mDixon Quant: Multi-echo two-point Dixon Quant
1H-MRS: Hydrogen Proton Magnetic Resonance Spectroscopy
TE: Echo Time
31P-MRS: - Phosphorus Magnetic Resonance Spectroscopy
αNTP/TP: Nucleotide Triphosphate to Triphosphate Ratio
Ktrans: Intravascular to Extravascular Interstitial Transport Coefficient
Ve: Extravascular Cell Volume Fraction
Vp: Plasma Volume Fraction
GdDTPA-BMA: Gadolinium Diethylenetriamine Pentaacetic Acid Bismethylamide
EES: Extracellular Extravascular Space
LSM: Liver Stiffness Measurement
AST: Aspartate Transaminase

## SUPPLEMENTARY MATERIAL

PRISMA checklist is available as supplementary material on the publisher’s website along with the published article.
